# A simple bypass assay for DNA polymerases shows that cancer‐associated hypermutating variants exhibit differences *in vitro*

**DOI:** 10.1111/febs.16936

**Published:** 2023-08-29

**Authors:** Gilles Crevel, Stephen Kearsey, Sue Cotterill

**Affiliations:** ^1^ MCS St George's University London UK; ^2^ Department of Biology University of Oxford UK

**Keywords:** DNA polymerase epsilon, DNA replication fidelity, polymerase‐associated hypermutation

## Abstract

Errors made by DNA polymerases contribute to both natural variation and, in extreme cases, genome instability and its associated diseases. Recently, the importance of polymerase misincorporation in disease has been highlighted by the identification of cancer‐associated polymerase variants with mutations in the exonuclease domain. A subgroup of these variants have a hypermutation phenotype in tumours, and when modelled in yeast, they show mutation rates in excess of that seen with polymerase with simple loss of proofreading activity. We have developed a bypass assay to rapidly determine the tendency of a polymerase to misincorporate *in vitro*. We have used the assay to compare misincorporation by wild‐type, exonuclease‐defective and two hypermutating human DNA polymerase ε variants, P286R and V411L. The assay clearly distinguished between the misincorporation rates of wild‐type, exonuclease dead and P286R polymerases. However, the V411L polymerase showed misincorporation rate comparable to the exonuclease dead enzyme rather than P286R, suggesting that there may be some differences in the way that these variants cause hypermutation. Using this assay, misincorporation opposite a templated C nucleotide was consistently higher than for other nucleotides, and this caused predominantly C‐to‐T transitions. This is consistent with the observation that C‐to‐T transitions are commonly seen in DNA polymerase ε mutant tumours.

## Graphical abstract

We have developed a bypass assay to rapidly determine the tendency of a polymerase to misincorporate *in vitro*. We have used the assay to compare misincorporation by wild‐type and exonuclease‐defective enzymes with the two most commonly occurring cancer‐associated hypermutating human DNA polymerase ε variants, P286R and V411L. The assay can distinguish between polymerases with different error rates and also suggests differences between the two cancer‐associated variants.

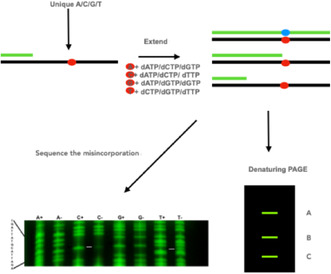

**Abbreviations**
dNTP: deoxynucleotide triphosphate
MMR: mismatch repair
POLE: DNA polymerase epsilon

## Introduction

Accurate synthesis by the replicative DNA polymerases is vital for the maintenance of genomic stability. 98–99% of this synthesis is carried out by DNA polymerases δ and ε, which have a high degree of accuracy [1] (∼ 1 in 10^−4^ to 10^−6^ from *in vitro* measurements), the rest being carried out by DNA polymerase α [2], which is slightly less accurate (∼ 1 in 10^−3^ to 10^−4^). It is thought that DNA polymerase ε carries out the bulk of the leading strand synthesis, while DNA polymerase δ acts predominantly, although not exclusively, on the lagging strand. The main reason for the increased accuracy of DNA polymerases δ and ε over polymerase α is their possession of a 3′‐5′ exonuclease activity, which is able to proofread and remove nucleotides which have been incorrectly incorporated. The exonuclease active site is in the largest subunit for both DNA polymerases δ and ε [3, 4, 5, 6, 7]. This subunit also contains a separate polymerase active site, and the nascent strand terminus moves ∼ 40 Å from the polymerase to the exonuclease site for proofreading. Loss of proofreading ability leads to a higher rate of synthesis errors *in vitro* and also *in vivo*, in both *Saccharomyces cerevisiae* and mice. [1, 8, 9, 10].

Recently, mutations have been identified in the pol/exo subunit of both DNA polymerase δ and ε that appear to be causative for the generation of tumours (reviewed in [11, 12, 13]). These were originally seen in colon and endometrial cancers but, more recently, have also been reported in other cancers. Many of the mutations studied so far have been in the exonuclease domain of the pol/exo subunit, and so their effects could be explained by the lack of exonuclease activity raising the misincorporation rate higher than can be handled by downstream mechanisms [particularly mismatch repair (MMR)]. However, for some mutations, the misincorporation observed *in vivo* is higher than that observed for an exonuclease dead mutant. This suggests that factors other than the lack of exonuclease activity must be responsible for the high mutation rates observed by these polymerases.

Many factors could be responsible for the observed hypermutation. These could be integral to the polymerase itself (e.g. changes in rate or processivity of synthesis) or could be due to factors only occurring *in vivo* (e.g. change in interaction with other proteins or due to handover to polymerases with lower fidelity). When studying the mechanisms of mutant polymerases, it is useful to know which of these possibilities is responsible for the high error rate, as this will give insight into how high replication fidelity is normally achieved.

**Fig. 1:**
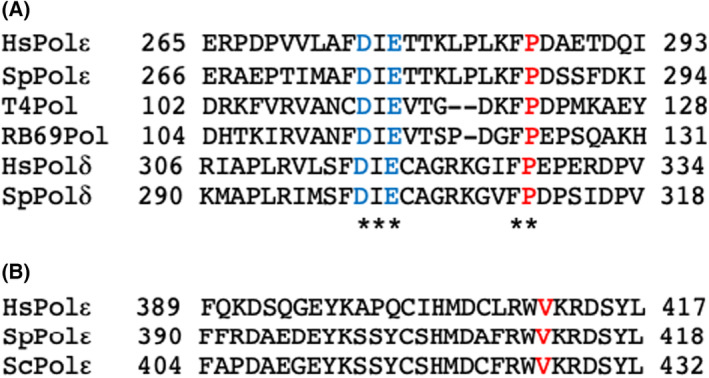
Comparison of the sequences of various polymerases in the region of the P286R and V411L mutations. (A) Conservation of the region around P286 in human polymerase ε. The conserved P is highlighted in red for human (P286), *S. pombe* (P287) and *S. cerevisiae* (P301) ε polymerases and human (P327) and *S. cerevisiae* (P332) δ polymerases. The conserved D and E residues which form part of the exonuclease active site are highlighted in blue. The asterisks show residues conserved between all polymerases compared here. (B) Conservation of the region around V411 in human polymerase ε. The conserved V is highlighted in red for Human (411), *S. pombe* (411) and *S. cerevisiae* (426) ε polymerases. This amino acid is not conserved in the δ polymerase family (not shown).

P286R and V411L are the two DNA Polymerase ε (POLE) variants that are the most frequent somatic variants associated with hypermutated cancer cells [11]. P286 is highly conserved in ε polymerases from a wide range of organisms, and also in DNA polymerase δ and phage polymerases (Fig. 1A). *S. cerevisiae* and *Schizosaccharomyces pombe c*ells expressing P286R also show a dramatic hypermutation phenotype [11, 14]. *In vitro* exonuclease assays show that an N‐terminal fragment of the human DNA polymerases ε catalytic subunit carrying the P286R mutation shows no exonuclease activity, while similar assays using the *S. cerevisiae* holoenzyme equivalent to P286R reported reduced but significant activity [14, 15]. V411 is also widely conserved in ε polymerases (Fig. 1B), but not in other polymerases, even those as closely related as δ polymerases. *In vitro* exonuclease assays suggest that human V411L retains a low level of activity [15]. Curiously, despite the high mutation rates in human cells, the *in vivo* mutation rate of the *S. cerevisiae* variant equivalent to V411L is similar to that of the wild‐type enzyme [16].

Here, we present a bypass assay to compare the *in vitro* misincorporation tendencies of wild‐type and mutant polymerase variants, where omission of a specific nucleotide forces the polymerase to misincorporate or generate an indel to synthesise a full‐length product. Application of this assay to analyse misincorporation for the P286R and V411L variants of the human DNA polymerases ε holoenzyme are consistent with recent results suggesting that these two variants may produce misincorporation via different routes.

## Results

### Wild‐type human DNA polymerase ε has limited ability to bypass a templated A nucleotide when dTTP is not available *in vitro*

**Fig. 2:**
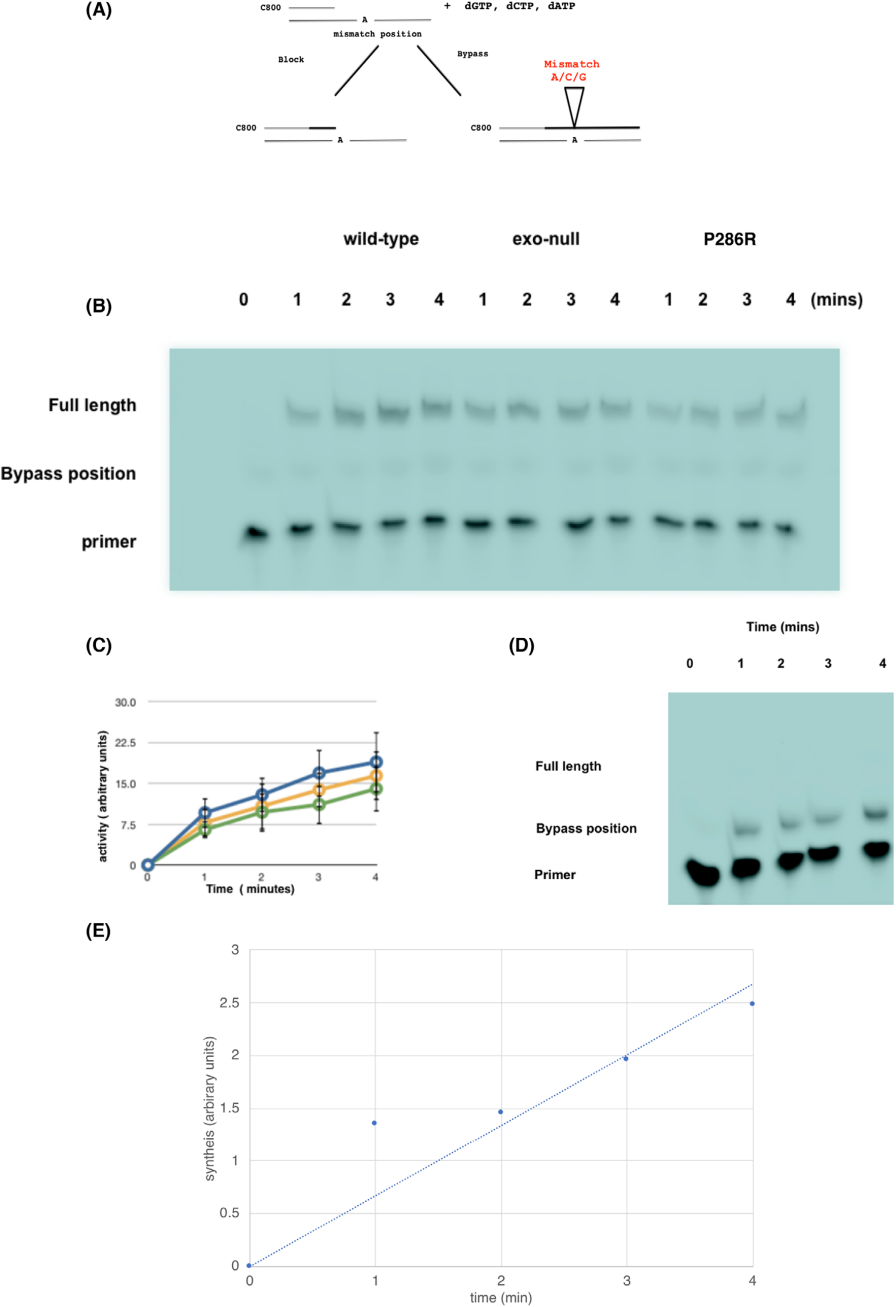
Bypass assay overview and results. (A) Basis of the bypass assay. The polymerase is provided with a C‐800 labelled template that has one of the bases at a single position, (in this case an A—marked as the mismatch position). The enzyme can only proceed to the end of the template if a misincorporation occurs; therefore, the ratio of the full length to the blocked template will give a measure of the bypass frequency and the ability of the polymerase to misincorporate. The conditions of the assay are arranged such that there is an excess of substrate compared with the enzyme; if the enzyme dissociates, it is more likely to bind to a new template than rebind to the original. The assay therefore represents the ability of an enzyme to misincorporate without dissociation from the template. (B) Representative gel comparing the synthesis by wild‐type, exo‐null and P286R POLE variants at 0, 1, 2, 3 and 4 min on template A1 in the presence of all four deoxynucleotide triphosphates (dNTPs). The positions of the unreplicated primer, the bypass position and the full length are marked. The faint band at the position of the bypass on this gel is due to xylene cyanol which coincidentally runs at the same position and so serves as a good indicator of the position of the template blockage. It can be distinguished from the DNA bands for quantitation purposes due to its different emission wavelength. This was repeated for each of the templates used for bypass and comparable results were obtained in all cases (*n* = 21). (C) Reactions similar to the one shown above were carried out for all templates and the results were combined to calculate the relative activity of the enzyme preparations used. In all bypass experiments, the amounts of exo‐null and P286R enzymes were adjusted so that the enzyme activity present in each reaction was comparable. The data shown are representative of three independent experiments for each enzyme. Results for the wild‐type enzyme are shown in blue, for the exo‐null in green and for P286R in yellow. The error bars show standard deviation. (D) Representative gel showing synthesis of the A1 template by the wild‐type DNA polymerase ε in the presence of only dCTP, dGTP and dATP after 1, 2, 3, and 4 min of reaction. The positions of the full length, bypass position and unreplicated primer are shown. Although this represents data from a single experiment, this analysis was routinely carried out for all experiments for all templates to ensure continued enzyme activity throughout the time course of the experiment (*n* = 28). (E) Quantitation of the results from (D) to show increasing synthesis of the stalled product with time. Although this represents data from a single experiment, this analysis was routinely carried out for all experiments for all templates to ensure continued enzyme activity throughout the time course of the experiment (*n* = 28).

One measure of intrinsic fidelity is to determine how easily a polymerase can bypass a base in the template if the complementary deoxynucleotide triphosphate (dNTP) is not available. This approach has been used previously with DNA polymerases ε looking at rapid reaction kinetics using stopped‐flow measurements [8] and also to determine the dissociation frequency of yeast DNA polymerases ε during the transfer of the primer end between the polymerase and exonuclease sites [17]. We therefore designed a template that had only a single A nucleotide present in the sequence, and in which the primer strand was labelled with the infrared dye C800 (selected because it can be analysed by infrared scanning in a quantitative way). The basis of the assay is shown in Fig. 2A. In the assay, there is a high ratio of primer/template to polymerase, so if the polymerase dissociates at the bypass position, it is unlikely to reassociate with the stalled product. In this way, the full‐length product is likely to reflect misincorporation by the polymerase at the bypass position, followed by continued synthesis in the absence of dissociation.

Synthesis carried out on this template by the wild‐type enzyme (purified as described in Materials and methods) with a full complement of dNTPs gave complete synthesis of the template with increasing yield with time (Fig. 2B,C), whereas synthesis carried out in the absence of dTTP showed increasing synthesis with time, but most synthesis was stalled at the position of the single A base (Fig. 2D,E).

### The exonuclease dead and P286R variants bypass a templated A with higher frequencies than the wild‐type enzyme

This encouraged us to use this assay to compare the *in vitro* bypass rate of the wild‐type enzyme with exonuclease dead and P286R mutants.

**Fig. 3:**
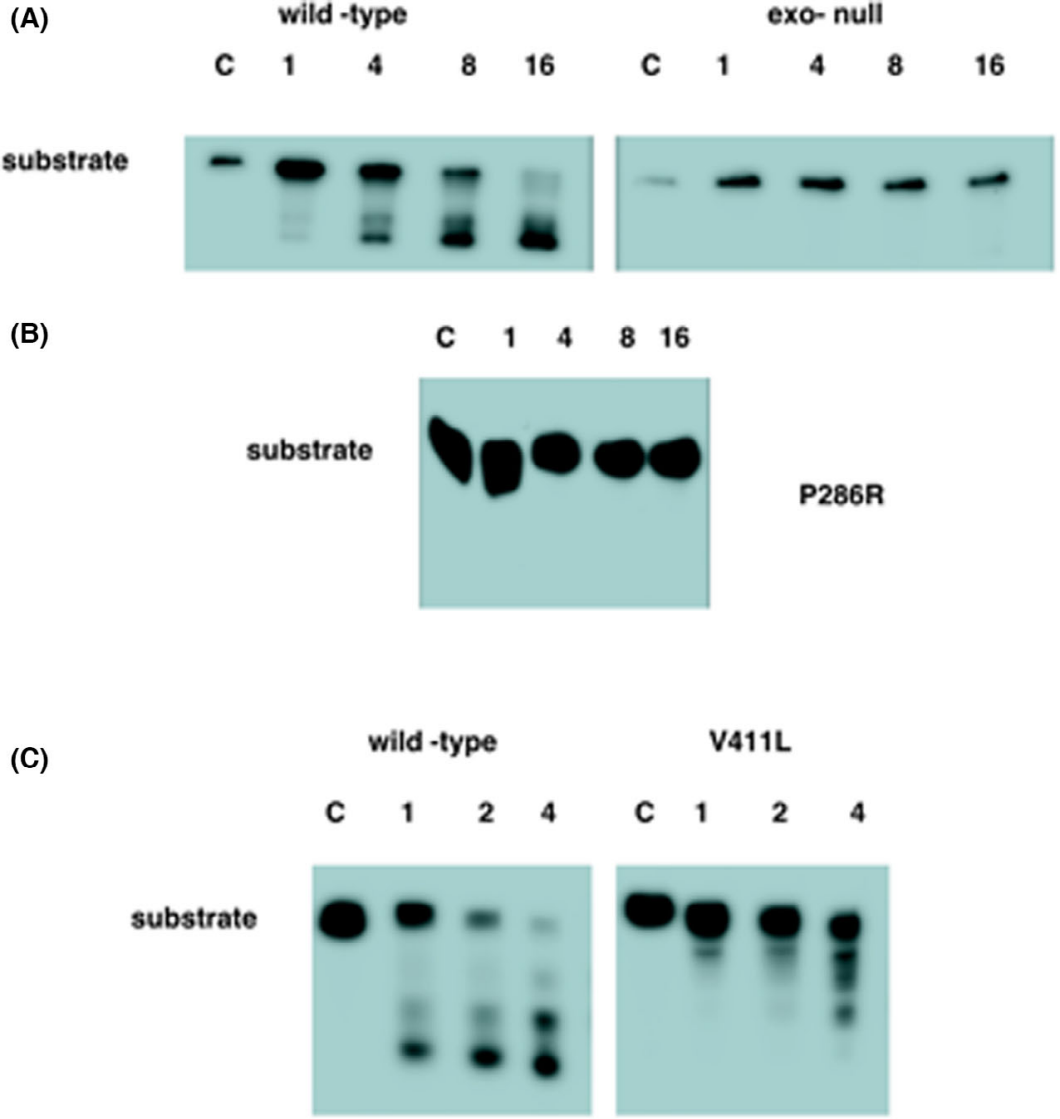
Representative gels showing exonuclease assays for purified Polymerase ε variants. (A) Exonuclease rates for wild‐type and exo‐null enzymes. C is the unreacted substrate and 1, 4, 8 and 16 are the time in minutes at which the time points were taken. (B) The reaction for the P286R variant from the same experiment. In this case, the samples have been overloaded and overexposed to check for residual exonuclease activity. (C) A comparison of wild‐type and V411L variants. C is the unreacted substrate, and in this case, time points were taken at 1, 2 and 4 min to allow a more accurate estimation of the level of exonuclease of V411L. Percentage activity for 411 was obtained by repeating the assay three times and scanning the gels produced on a Licor Odyssey machine.

We therefore introduced mutations into the large subunit of DNA polymerase ε to generate exonuclease dead [where the exonuclease catalytic residues have been mutated (D275A E277A)] and P286R variants. An analysis of the exonuclease activity of the wild‐type, and the exonuclease dead and P286R variants (Fig. 3A,B) showed that neither the exonuclease dead nor P286R mutants showed exonuclease activity *in vitro*. This is consistent with previous observations from *in vitro* studies using an N‐terminal fragment of the human enzyme [15]. This suggests that previously observed differences in exonuclease activity between the *S. cerevisiae* and human P286R variants were not caused by the use of a truncated form of the human enzyme and may indicate that the budding yeast enzyme is affected differently by the mutation.

**Fig. 4:**
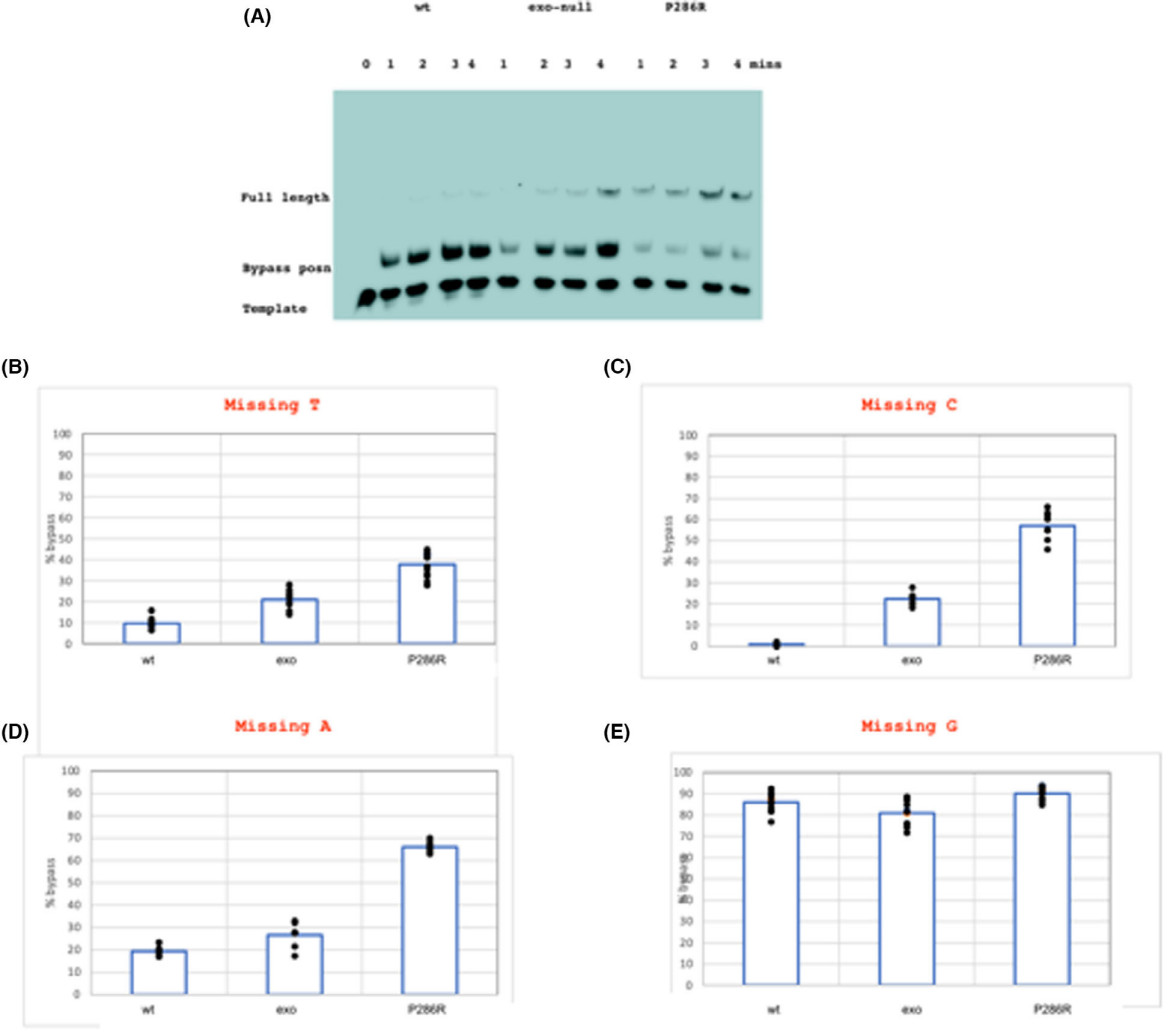
(A) Synthesis of A1 template by wild‐type, exonuclease dead and P286R variants in the presence of only dCTP, dGTP and dATP after 1–4 min of synthesis. 0 is the unreplicated template The positions of the full length, bypass position and unreplicated primer are shown. (B) Percentage bypass rates on the A1 template (i.e. missing T) for the wt, exonuclease dead and P286R variants in the presence of only dCTP, dGTP and dATP. (C) Percentage bypass rates for the G template (i.e. missing C) for the wt, exonuclease dead and P286R variants in the presence of only dATP, dGTP and dTTP. (D) Percentage bypass rates for the T template (i.e. missing A) for the wt, exonuclease dead and P286R variants in the presence of only dCTP, cGTP and dTTP. (E) Percentage bypass rates for the C1 template (i.e. missing G) for the wt, exonuclease dead and P286R variants in the presence of only dCTP, cATP and dTTP. For all the above experiments, the data shown are representative of three independent experiments. each with four time points. Dots represent individual data points. Comparing the results between pairs of enzymes (wt vs exo, exo vs 286, wt vs 286,) using the Student *t*‐test gave *P* < 0.001 for A, G and T templates. For the C template, *P* = 0.025 for the wt vs exo comparison and < 0.001 for exo vs P286R and wt vs P286R.

An example gel for the bypass analysis with these three variants is shown in Fig. 4A. The exonuclease dead enzyme showed a higher bypass rate than the wild‐type, and P286R showed a higher rate still. Quantitation of the results for multiple experiments using template A1 is shown in Fig. 4B.

**Fig. 5:**
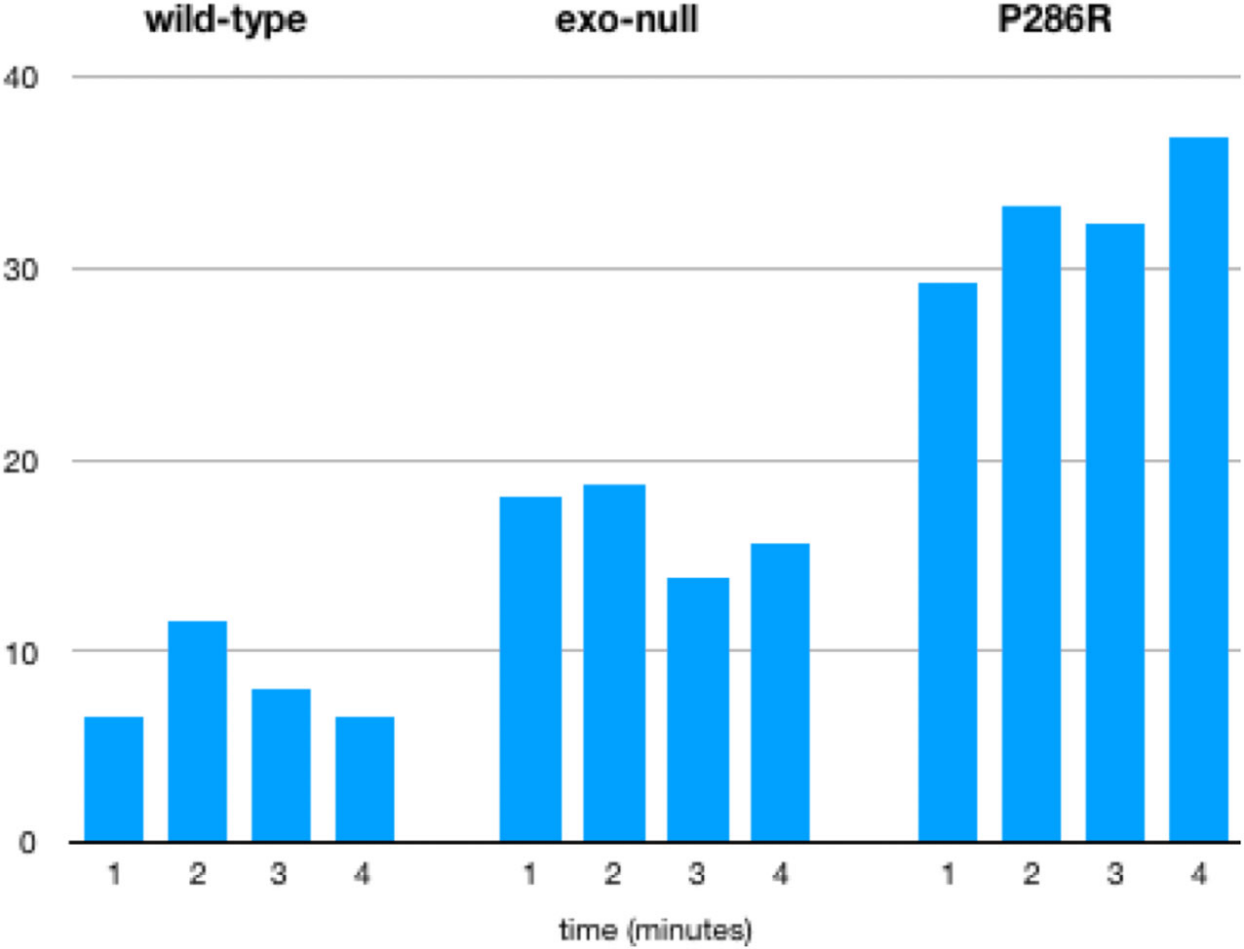
Relative bypass rates do not increase with time for the wild‐type, exo‐null and P286R variants under the conditions of the assay. The time in minutes is shown on the *X*‐axis and the percentage bypass on the *Y*‐axis. This shows the results from one assay with the A1 substrate, but this analysis was routinely carried out for all experiments on all templates used for bypass and a similar lack of correlation between bypass rate and time was observed (*n* = 28).

Comparison of the bypass rate at multiple time points (Fig. 5) showed that the percentage of bypass did not increase with time and provided that a significant fraction of the template remained unreplicated. This implies that the stalled product, once generated, is not eventually extended to full length, suggesting that the full‐length product is likely to be due to a misincorporation event which is extended by the same polymerase without dissociation. Therefore, in all subsequent experiments, bypass rates presented are an average of the bypass at each time point for each enzyme.

These results suggest that this assay is sufficiently sensitive to detect differences in the bypass rates between polymerases with different fidelities. In addition, since the bypass rate depends on the ratio of full‐length synthesis to total synthesis, the measurement is not significantly affected by the absolute enzyme concentration. This reduces measurement errors between different enzymes. The data further suggest that the hypermutation phenotype of the P286R mutant is at least in part amenable to *in vitro* analysis, as it has higher bypass rate in the *in vitro* analysis than the exonuclease dead mutant.

### Differences in bypass efficiency for human POLE mutants are also observed for bases other than A

Similar experiments were then carried out to determine whether differential bypass by wild‐type, exonuclease dead and P286R polymerases could also be observed for the three other nucleotides. For both G (Fig. 4C) and T (Fig. 4D) templates, a similar trend was observed although the absolute bypass rates varied for different nucleotides. For the C1 template, however, a very high bypass rate was observed even with the wild‐type enzyme (Fig. 4E). This shows that differences in misincorporation between, wild‐type, exonuclease dead and P268R enzymes are not specific to bypass of an A residue.

### Bypass rates of ‘single C’ templates are influenced by the context of the base and the concentration of dNTPs in the reaction

The very high rate of bypass on the C1 template was surprising, especially in the case of the wild‐type enzyme, but was entirely reproducible using different independent batches of nucleotides and several independently synthesised templates carrying the same sequence. Comparison of the results observed using the A1, G and T templates suggested that although the relative abilities of the different POLE enzymes to misincorporate opposite the single base were conserved, there were differences in absolute bypass rates between different bases. This could be intrinsic to individual bases; however, the absolute bypass rate could also be influenced by the context of the bypassed base.

**Fig. 6:**
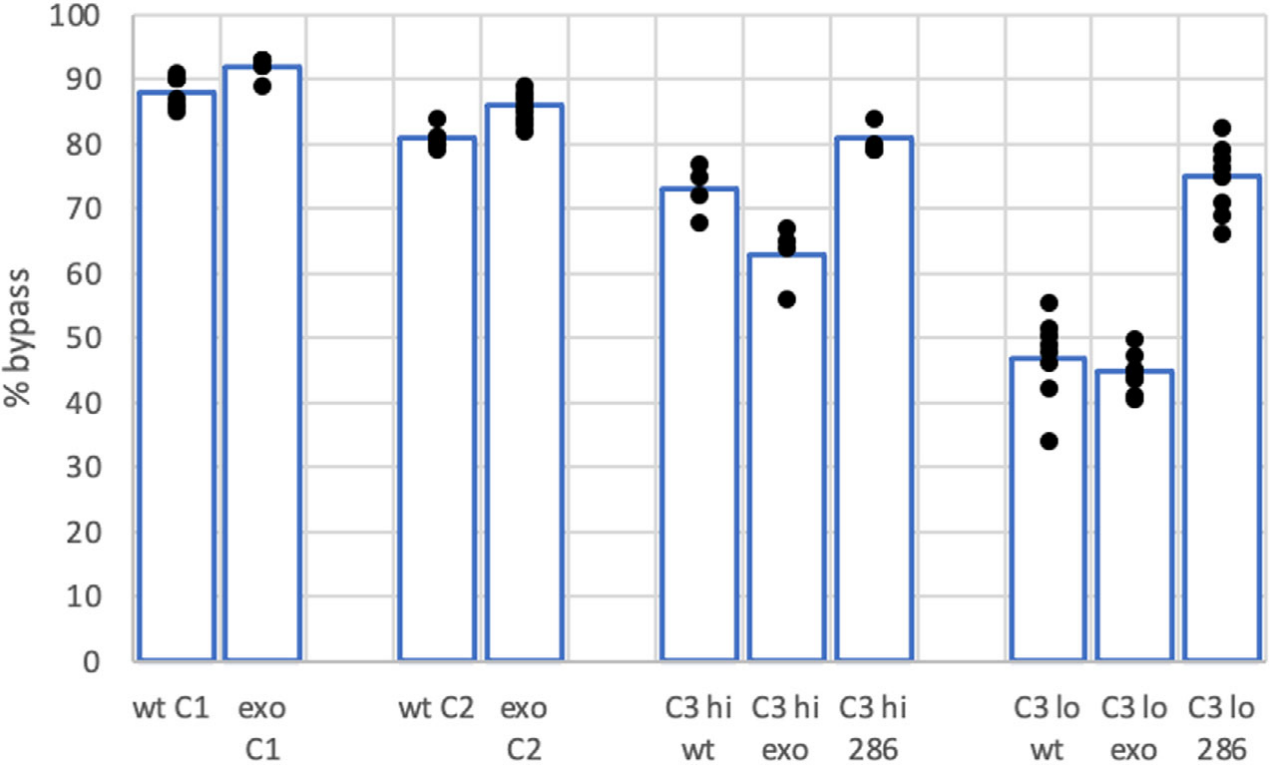
Comparison of bypass rates for templates C1, C2 and C3 at 200 μm (hi) or 80 μm (lo) dATP, dCTP and dTTP. The *Y*‐axis shows the % bypass rate. The *X*‐axis shows the template and the variant used for the assay: wt—wild‐type, exo—exonuclease dead and 28—P286R. The data shown are representative of three independent experiments. Comparing the results between pairs of enzymes, using the Student *t*‐test gave *P* ≤ 0.01 for all comparisons (exo vs wt, exo vs P286R, wt vs P286R, C1 vs C2 for wt and exo, C1 vs C3 for wt exo and P286R, and high vs low dNTPs for wt, exo and P286R).

We therefore determined how the ‘single C’ bypass rate was affected by the context in which the C is embedded. We synthesised a template (C2) in which the immediate environment of the single C residue was altered 4‐bp upstream and downstream of the C. This change did not affect the base composition of the template but made the two bases either side of the C more AT rich (GGAAGTCGTATTA to GGGTAACTTAGTA). For this template, the bypass rate was slightly reduced, (Fig. 6), but still remained much higher than for the other nucleotides. We also made an additional C template (C3) where the downstream sequence was the original template, but the upstream template was more substantially altered. In addition, the single C was moved further away from the primer terminus by the insertion of eight nucleotides (TTGAAGTAGTGAGATGGAAGTC to TTGAAGTAGTGAGATGGAAGTGTAGATTAC). This template also produced bypass levels for all three enzymes that were slightly lower than those for the other C templates (Fig. 6), but again bypass levels, particularly for the wild‐type enzyme, were higher than for other bases.

Another factor that could influence the amount of bypass is the concentration of the dNTPs present in the reaction. The presence of an increased concentration of dNTPs encourages misincorporations *in vivo* in a variety of organisms and this is likely to be at least partly due to direct effects on the polymerase [18]. Figure 6 shows that reducing the concentration of dNTPs in the reaction by 60% (to 80 μm) reduced bypass levels quite significantly for the C3 template. However, bypass levels still remained higher for C3 at low nucleotide concentrations than for the A1, G and T templates at higher dNTP concentrations. Again, this was particularly noticeable for the wild‐type enzyme.

One curious feature that we observed consistently for bypass on all C templates at all dNTP concentrations was that the exonuclease dead enzyme had a similar bypass rate to the wild‐type enzyme although for the C3 template, the P286R read through is clearly higher. These results suggest that it is possible to optimise the bypass assay for a ‘C’ template so that differences in bypass can be observed between a wild‐type enzyme and a hypermutating enzyme, but in its present format, bypass of a single C may not be able to distinguish a wild‐type enzyme from one that has only lost exonuclease activity.

### Bypass rates at ‘physiological’ dNTP concentrations are raised for P286R

**Fig. 7:**
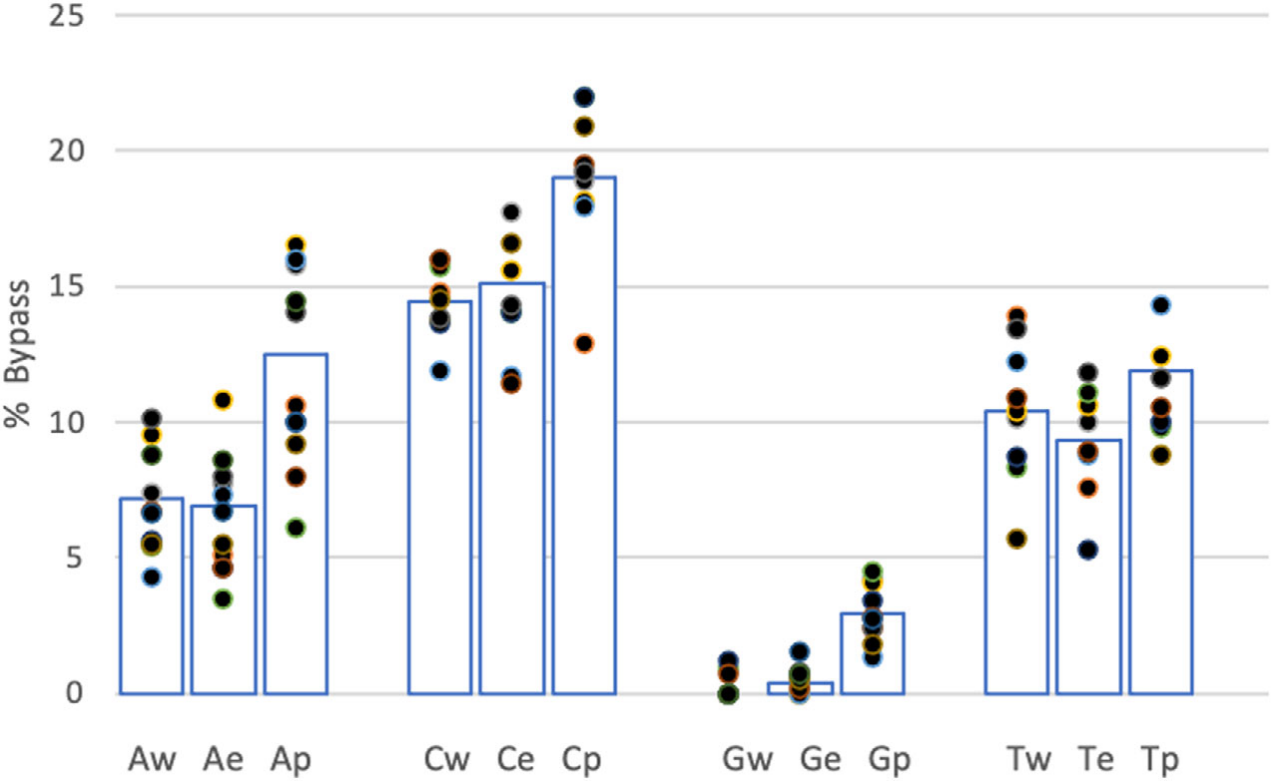
Bypass rates for wild‐type, exonuclease dead and P286R at estimated physiological deoxynucleotide triphosphate concentrations and ratios in nontransformed cells. The *Y*‐axis shows the % bypass rate. The *X*‐axis shows the templated base (A1, C1, G and T) and the polymerase variant w—wild‐type, e—exonuclease dead, p—P286R. The data shown are representative of three independent experiments. Comparing the results between pairs of enzymes using the Student *t*‐test showed that differences between the wt and exo enzymes were not significant, however, for all comparisons of exo vs P286R and wt vs P286R < 0.001.

All previous assays were carried out at dNTP concentrations that were in the range of values measured for tumour cells, but higher than those seen for nontransformed cells. To determine how lower dNTP concentrations affected the observed bypass rate, we repeated the bypass experiment using dNTP concentrations estimated for nontransformed cells (dATP 25 μm; dGTP 5.2 μm; dCTP 30 μm; and dTTP 35 μm) [19]. Figure 7 shows that under these conditions, the differences between the wild‐type and exonuclease dead enzyme were not detectable. The bypass rate for P286R still remained higher than that for the other two variants, but the differences were less pronounced.

### Bypass on the C3 template predominantly causes a G to A (C to T) misincorporation

**Fig. 8:**
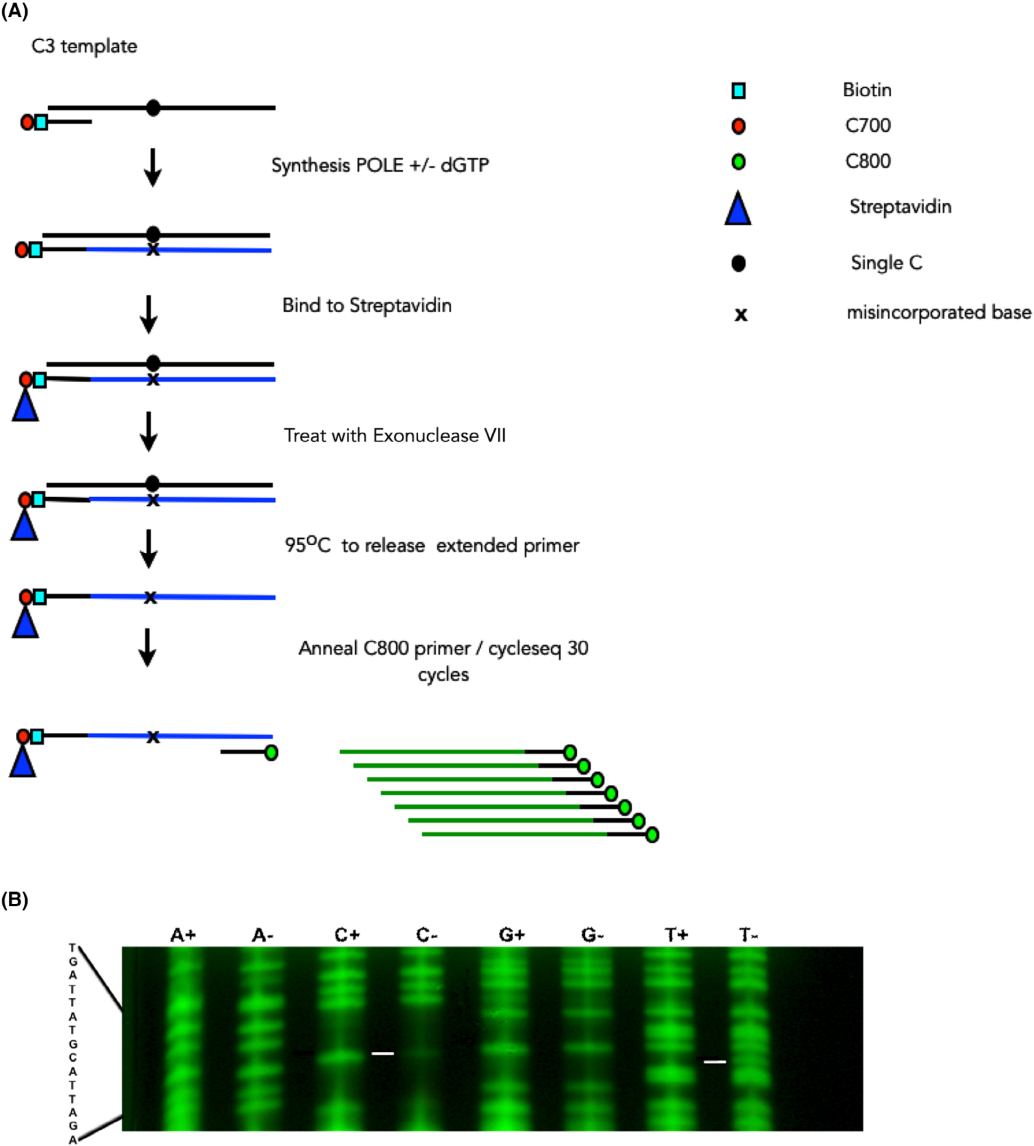
Determination of POLE misincorporation preferences on the C3 template. (A) Scheme for sequencing of the products of POLE synthesis. The presence of the C700 label in the biotin‐labelled primer allows verification of the extent of bypass by POLE in the initial reaction. The exonuclease VII step is designed to remove unannealed template and unextended primer. The heating step is designed to allow removal of the template strand. (B) Results from the cycle sequencing following the bypass assay using the C3 template and the P286R variant. The equivalent nucleotide lanes are run next to each other to make it easier to see changes. A C G T shows the chain terminator used for sequencing, + is the analysis of the bypass reaction containing all four nucleotides, and − is the analysis of the bypass reaction missing dGTP from the extension mix. The expected sequence is shown at the left‐hand side of the gel. The lines in the gel show the position of a visible change between the reaction containing all four nucleotides and the one missing a G in the extension mix, comprising a reduction in the C‐lane and a new band appearing in the T‐lane. This shows the result of a single gel, but the experiment was repeated six times and produced the same result each time.

It still remained a formal possibility that some contamination in either the template or the dNTP mix was responsible for the high bypass rate with the C templates. Ganai *et al*. [17] showed that for purified yeast POLE holoenzyme, very low levels of dGTP (0.34–0.68 μm) were enough to support some synthesis. In order to determine whether this was the case, we developed a protocol to sequence, the DNA that had been synthesised by the DNA polymerases ε variant enzymes. This protocol allows us to use very small amounts of synthesised product and so would be applicable to reactions where the bypass is less efficient. The scheme uses cycle sequencing to determine the base that had been incorporated at the single C position. The scheme is shown in Fig. 8A and incorporates steps to remove nonreacted or partially extended primers, and also as much as possible of the original template, all of which interfere with the cycle sequencing reaction if they remain in the solution at a significant level.

Figure 8B shows the results of a sequencing reaction with the synthesis products of P286R on the C3 template. C3 was used as the template as the single C was far enough away from the sequencing primer to identify the incorporated nucleotide clearly. At the position on the template where the C is incorporated in a reaction containing all 4 dNTPs (+), the bypass reaction (−) shows only trace incorporation of C. A low level of C incorporation would be generated if traces of the original template and primer remained following the exonuclease reaction and heat treatment. These could anneal, and, during the cycle sequencing reaction, would generate a C at this position. Instead, a new band appears in the T track suggesting that if P286R is unable to incorporate a dG in the template opposite a dC, it preferentially incorporates a dA. No new bands are visible in other lanes, but we cannot rule out the possibility that bands are present but below the level of detection. Similar results were obtained with both the exonuclease dead and the wild‐type enzymes. Skipping (deletion) at the bypass position does not occur as the +/− bands are in alignment on both sides of the bypass position.

This confirms that the high bypass rate on the C template cannot be explained only by a contamination of either the template or the dNTP mix. It further suggests that there is some specificity to the dNTP misincorporated during the bypass. The apparent ease with which DNA polymerase ε incorporates dA when there is a template dC may account for the frequency of C > T transitions in yeast strains expressing an exonuclease‐defective DNA polymerase ε in the background of MMR deficiency [20].

### V411L does not show an increased bypass rate compared with the exonuclease dead variant

Another common cancer‐related DNA polymerase ε variant that has been identified as a hypermutator is V411L. The location of this mutation is further away from the exonuclease active site than P286R. In addition, evidence from studies on human cancers and cancer cell lines carrying different POLE mutations suggests that there may be some differences in the mutational signatures of P286R and V411L *in vivo* [21, 22, 23]. V411L has also been shown to retain some exonuclease activity when the N‐terminal domain of the large subunit was assayed *in vitro* [15].

**Fig. 9:**
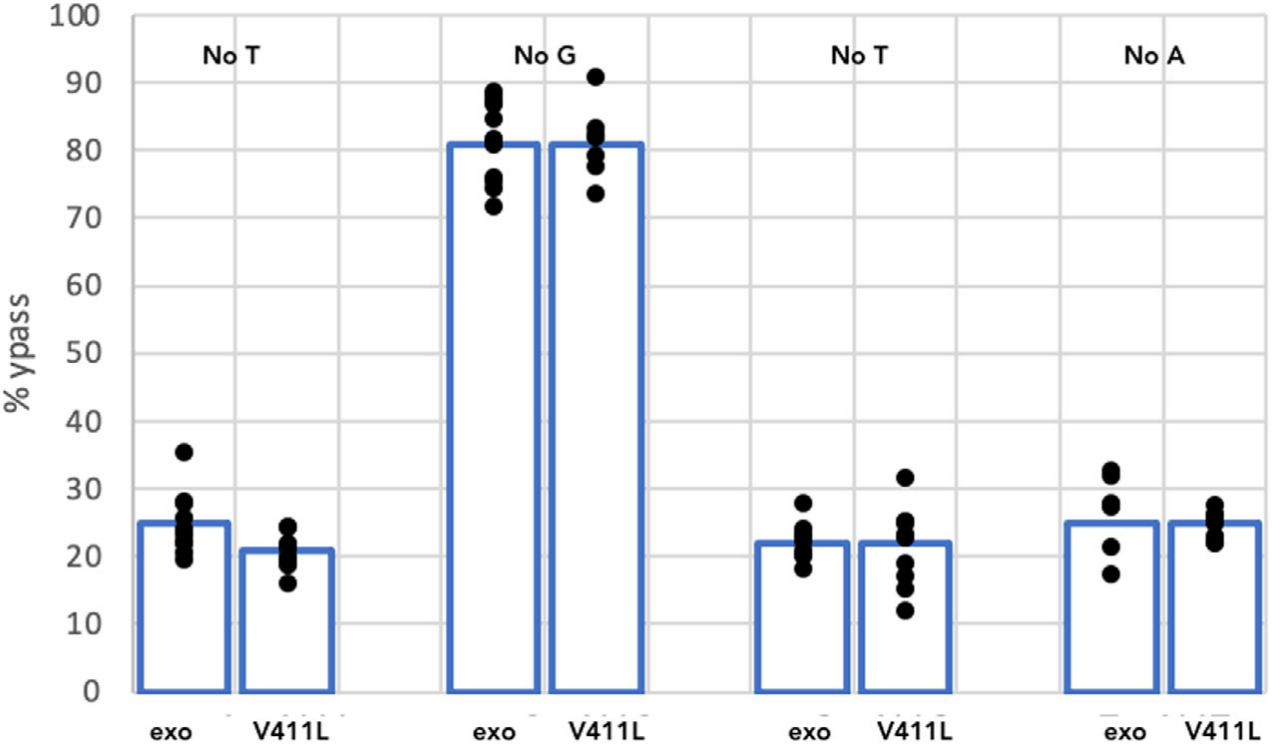
Percentage bypass rates for exonuclease dead and V411L for the A1, C1, G and T templates under the standard reaction conditions (200 μm of each nucleotide). The *Y*‐axis shows the % bypass rate. The missing base is shown above the relevant lanes (No T = A1 template, No C = G template No G = C1 template NoA = T template). Exo shows data for the exonuclease dead and V411L for the V411L variants, respectively. The data shown are representative of three independent experiments. Comparing the results between exo and V411L using the Student *t*‐test for each template showed that differences between the enzymes were not significant.

We were interested in determining whether we could detect an increased misincorporation frequency for this variant *in vitro*. We therefore introduced this mutation into the large subunit of polymerase ε. Consistent with previous results, we saw that the holenzyme containing the mutation retained a low level of exonuclease activity (Fig. 3). We then analysed the ability of the V411L mutant enzyme to perform bypass synthesis for A1, C1, G and T templates. Although V411L is known to be a hypermutator in human cells, the bypass frequency appeared more similar to that of the exonuclease dead mutation than that of P286R (Fig. 9). This is consistent with *in vivo* observations which suggest that the mechanism causing hypermutation in V411L may differ from that in P286R.

## Discussion

We have developed a rapid bypass assay to measure the likelihood of polymerase misincorporation using templates containing a single A, C, G or T when the required complementary nucleotide is not present in the polymerisation reaction. We have used the assay to measure the bypass frequency associated with a 4 subunit wild‐type human DNA polymerase ε and compared this to the bypass observed for a variant of the polymerase that has been mutated to inactivate the exonuclease activity, and two other variants that have been altered to contain mutations commonly detected in colon cancer‐associated polymerases.

Using this assay, it is possible to clearly detect a measurable difference in misincorporation ability between purified DNA polymerase ε variants with exonuclease domain mutations. Bypass rates can be determined for a single A G or T in the template. However, the assay does not provide a quantitative measure of *in vivo* fidelity, as the conditions used for the assay are not conditions that would usually be experienced by the enzyme in a cellular setting. In addition, the precise reaction conditions also affect the amount of bypass that is obtained.

The observed bypass rate in our experiments varies depending on the identity of the templated bases. For instance, the observed bypass ratio of exonuclease dead, P286R and V411L, compared with wild‐type all appear much higher for G than for A, T and C. This could suggest that the wild‐type enzyme more accurately incorporates opposite a templated G. However, it is possible that the accuracy observed for the G template could be specific for the particular template and conditions used in the assay. This would be consistent with the data that we obtained using C templates which suggest that the bypass rate might vary depending on the context of the base, and also the concentration of dNTPs that are used in the assay. Similar effects of chromosome context and dNTP concentration have been reported previously (reviewed in [24]). The concentrations of dNTPs used for the bypass assay are comparable with the concentrations routinely used for *in vitro* polymerase assays and are in a similar range to those seen in tumour cells. However, they are higher than previously measured cellular dNTP levels, which range between ∼ 5 and ∼ 40 μm depending on the nucleotide [19]. The dNTP levels also show differences between different cell types and cell cycle stages and in addition may be affected by subcellular location. When we carried out the bypass at ‘physiological’ concentrations, the differences between the variants were much reduced, with differences between the wild‐type and exonuclease dead variant no longer being detectable. The precise reasons for this are not clear; however, it may be related to the reduced rate of synthesis under these dNTP conditions increasing the fidelity of the polymerase reaction. The lower ratios of bypass observed under ‘physiological’ conditions suggest that higher concentrations of dNTPs are preferable for maximum sensitivity of the bypass reaction.

The observation that bypass rates are dependent on dNTP concentration complicates direct prediction of *in vivo* fidelity from the bypass assay. Direct quantitation of *in vivo* fidelity are further complicated as some component of the *in vivo* measured fidelity may not be intrinsic to the polymerase, but due to another factor such as a corrupted interaction of the polymerase with other cellular components or other factors, such as polymerase switching. Although not a direct measure of *in vivo* fidelity, the relative bypass rate measured by this assay does give insight into the likelihood of a polymerase having a mutator phenotype *in vivo*. Further optimisation of the assay, by altering the template and the nucleotide concentration, should also increase the sensitivity of the assay to allow detection of smaller differences in misincorporation frequency and to determine whether the polymerase stalls pre‐ or postmisincorporation.

### Distinct behaviour of single C templates compared with single –A/G/T templates

The bypass assays using the ‘single C’ template showed two features that differed from the other single base templates. The first of these was that all C templates had a much‐increased level of bypass at the standard dNTP (200 μm) concentrations used for the assay compared with A, G and T templates. The C1 template has a TCG context around the C, whereas the other two templates have ACT (C2) and ACG (C3). TCG is one of the triplets associated with a high frequency of C > T transitions in POLE tumours [22], which could explain why this template had a slightly higher rate of bypass than the other templates. However, the bypass frequency was also high for the other two templates, so this cannot fully explain the high bypass rates observed on ‘single C’ templates.

Reducing the dNTP concentrations to 80 μm significantly reduced the bypass rate for the wild‐type and exonuclease dead enzymes, although the rate was still higher than that for the other bases at 200 μm. This suggests that under the conditions of the assay, POLE is more likely to misincorporate opposite a C base in the template than opposite any of the other bases. At this point, it is not clear why this should be the case. C is the only base that shows substantial methylation in humans, and therefore, it is tempting to speculate that the relaxed precision may be due to the need to accommodate the methylated variant. In this regard, it will be interesting to see whether higher C bypass rates are also found in species which do not show high levels of C methylation.

Sequencing of the DNA polymerase ε synthesised DNA strand from the C3 template showed that there was a very high tendency for an A to be incorporated by the enzyme in place of the G, that is misincorporation results in a C > T transition. A and G are both purines, and so this substitution would cause the minimum disruption to the double‐stranded structure produced. This substitution is also consistent with the observation that C‐to‐T transitions are commonly seen in DNA polymerase ε mutant tumours, particularly in a CG context [22], in yeast exonuclease‐defective DNA polymerase ε strains lacking MMR and in *S. pombe* expressing the equivalent mutation (in this case P287R) [25]. The choice to incorporate an A rather than any other nucleotide must be made at the level of the polymerase catalytic site since the same misincorporation is seen, not only for the wild‐type DNA polymerase ε, but also for the exonuclease dead and P286R variants. Neither exonuclease dead nor P286R possess exonuclease activity, so it would not be able to change the nucleotide added to the growing chain after incorporation.

The second and more surprising observation was that although the ‘single C’ templates and conditions used here clearly distinguish between a DNA polymerase ε wild‐type and P286R mutant, they do not allow a definitive distinction between wild‐type and exonuclease dead enzymes. For all C templates, the exonuclease dead enzyme showed a similar bypass rate compared with the wild‐type enzyme. The reasons for this are not clear; however, the wild‐type bypass frequency is increased to levels at least as high as that seen with other nucleotides for the exonuclease dead. It is therefore possible that, at least under the specific conditions of this assay, wild‐type DNA polymerase ε may have an impaired capability to remove a misincorporated base opposite a C in the template.

### Hypermutating mutants V411L and P286R behave differently in the bypass assay

We have also used the assay to compare the bypass rate associated with two mutations that are commonly seen in human tumours, P286R and V411L. These variants have been reported to show some differences in mutation signatures in patient samples and cell lines, such that SBS 10B and SBS10A are more predominant in V411L and P286R, respectively, and V411L may be less affected if MMR is inactivated [21, 22, 23]. In addition, our assays, and those reported by Shinbrot *et al*. [15], show that V411L retains some exonuclease activity, while P286R does not (Fig. 3). In contrast to P286R, V411L showed a bypass rate that was similar to the exonuclease dead. Although our data are not sufficient to draw a definitive conclusion about the cause of this difference, it is tempting to speculate that P286R and V411L might generate mutations using different mechanisms. This would not be surprising given their different locations. P286R is located at the edge of the exonuclease active site and structural analysis of the yeast enzyme suggested that the amino acid change blocks entry of the nascent DNA terminus to the exonuclease site [14, 26]. V411 is quite well separated from the exo site and lies in the junction region between the exo and polymerase site. It is therefore less likely to block entry to the exonuclease site. It does however lie in a region of the enzyme that could make contacts with the DNA substrate in addition to the active site contacts. Perhaps, the position of the V411L substitution makes the polymerase more likely to dissociate from the template as it transfers between the polymerase and exonuclease sites when the wrong nucleotide is incorporated. With the excess of template present in the assay, rebinding would be more likely to occur at a new primer–template than primer–template that is already partly extended and mismatched.

### Conclusion

We have developed an assay that allows us to rapidly measure the intrinsic ability of a purified polymerase to misincorporate nucleotides in the absence of other accessory factors. This assay should be useful to give an idea of the likely fidelity of mutations in purified polymerase enzymes and will be useful for rapid analysis of cancer‐associated mutants to guide routes for further study. Sequence analysis of the misincorporations will also yield insight into the relative contributions to mutation signatures of the properties of the polymerase and downstream repair‐related activities. Finally, the assay may also be useful as a tool for development of drugs that affect the accuracy of a polymerase, either positively or negatively.

## Materials and methods

### Manufacture of DNA synthesis substrates

Oligonucleotide primers labelled with C700 and C800, and where appropriate, also with biotin, were obtained from IDT and nonlabelled template oligonucleotides from Sigma. The substrates were made by mixing equal amounts of primer and template oligos (300 pm) in 20 mm Tris pH8, 10 mm KCl and 0.01 mm EDTA. Samples were heated to 100 °C for 15 min and then allowed to cool slowly to room temperature. The extent of annealing was assessed using native TBE polyacrylamide gels. In all cases, annealing of the labelled primer was estimated to be more than 99%. The oligonucleotides used in the assays are shown in Table 1.

**Table 1:** Oligonucleotides used in this analysis.

Oligonucleotide	Sequence
Single A1	TTGCCGTCGTGCGCTGGCCGTAGTCTTCGTCAACGTCGTGACT GGGAAAA
Single C	TTGAAGTAGTGAGATGGAAGTCGTATTA GTCAACGTCGTGAC TGGGAAA
Single G	TTACCATCATACACTAACCATGATCTTCATCAACGTCGTGACT GGGAAAA
Single T	AAGCCGACGAGCGCAGGCCGATGACAACGACAACGTCGTGAC TGGGAAAA
Single C2	TTGAAGTAGTGAGATGGGTAACTTAGTA GT CAACGTCGTGACT GGGAAA
Single A2	CGCTGGCCGTAGTCTTCCAACGTCGTGACTGGGAAAA
C700/800 primer	TTTTCCCAGTCACGACGTTG
Single C3	TTGAAGTAGTGAGATGGAAGTGTAGATTACGTATTAGT CAACGTCGTGACTGGGAAA
C800seq C	TTGAAGTAGTGAGATGG

### Cloning of wild‐type and mutant DNA polymerases

Clones for the wild‐type DNA polymerase ε subunits were a kind gift from the Hurwitz Lab [27]. The original p261 clone was tagged with a Flag epitope. Since we were interested in the properties of intact complexes rather than the isolated p261, the tag was removed from the large subunit and a Flag tag added to the p58 subunit. The exonuclease dead, P286R and V411L variants were obtained by carrying out point mutation to these clones using the QuikChange II Site‐Directed Mutagenesis Kit (Agilent, Santa Clara, CA, USA). The oligonucleotides used to produce these mutations are shown in Table 2. The changes introduced to make the polymerase devoid of 3′‐5′ exonuclease activity (exonuclease dead) were based on work reported from yeast and mice [9, 10].

**Table 2:** Nucleotide changes made to produce the human POLE variants used in this study.

Polymerase	Amino acid mutation	Nucleotide mutation
Wild‐type	n/a	n/a
Exonuclease dead	D275A E277A	a824c a830c
P286R	P286R	c857g
V411L	V411L	g1231t

### Purification of wild‐type and mutant polymerases

The DNA for the wild‐type and mutated sequences was introduced into the pFastBac vector (Thermo Fisher Scientific) and transfected into DH10Bac *Escherichia coli* in order to produce bacmids containing polymerase subunits. Selection of positive clones was carried out using blue/white selection. Clones for each of the four subunits were used individually to infect sf9 cells to produce baculovirus stocks.

To isolate polymerase complexes, Sf9 cells were infected with Bacmids for each of the four subunits simultaneously and grown for 72 h. Cells were harvested and the enzymes were purified as described [27] with a final glycerol gradient step to ensure that only intact holoenzymes were present. The purity of the polymerase enzymes was assessed after the glycerol gradient step by SDS/PAGE and Coomassie gel staining. Protein concentrations were calculated using serial dilutions of Bio‐Rad protein markers.

The polymerase activity of each prep was calculated using the A2 substrate in the presence of all four nucleotides.

### Bypass assays

Polymerase assays were carried out using templates as described above. 25 μL reactions contain; enzyme, 20 mm Tris pH 7.5, 10 mm magnesium acetate and 150 mg·mL^−1^ BSA together with 12 pm DNA template and the appropriate dNTP mix (200 μm of each nucleotide for the standard reaction, 80 μm for C1/C2/C3 comparison, and dATP, 25 μm; dGTP 5.2 μm; dCTP, 30 μm and dTTP 35 μm for reactions at ‘physiological’ concentrations). Reactions were incubated at 37 °C. 20 fm of wild‐type polymerase added to reactions to ensure that the template was always in large excess over enzyme. For all assays, the amount of each mutant polymerase was adjusted so that it had the same polymerase activity as the wild‐type enzyme. Samples were taken at 1, 2, 3 and 4 min for assays with dNTP concentrations at 200 and 80 μm and 2, 4, 6 and 8 min with ‘physiological’ dNTP concentrations. The reaction was stopped by the addition of denaturing gel loading buffer (Thermo Fisher) supplemented with EDTA. Samples were heated at 100 °C for 5 min to denature the strands and then analysed on 10% denaturing TBE gels containing 7 m urea. The gels were visualised using a Licor Odyssey CLx imaging system and where applicable quantitated using Licor software. In each case, the % bypass was calculated as:

$$\frac{\text{primer extended fully}}{\text{primer extended fully}+\text{primer extended to the bypass position}}.$$

### Exonuclease assays

These were carried out under the same conditions as the polymerase assays except that dNTPs were omitted from the assay, and the samples were run on 15% denaturing gels. The template used was A1 annealed to a version of the standard primer with an added mispaired G on the 3′ end. Where appropriate, the exonuclease activity was calculated by quantitation of band intensities using licor odyssey software (Lincoln, NE, USA) and calculating the total number of bases removed in each case.

### DNA sequencing

DNA sequencing was carried out using the Thermo Sequenase Cycle Sequencing Kit from (Applied Biosystems/Thermo Fisher Waltham, MA, USA). Briefly, oligonucleotides labelled with C700 and biotin were annealed to the appropriate templates as described. These were used as substrates for a polymerase reaction, and the reaction was allowed to proceed for 20 min. The reaction was stopped using 25 mm EDTA, and the products were purified by binding to streptavidin magnetic beads. To reduce background interference from oligonucleotides bound to the column that were unextended or not fully extended at the end of the reaction, the beads were subjected to digestion with exonuclease VII for 60–90 min at 37 °C. To reduce background interference from the original oligonucleotide templates that remained in the reaction, the reactions were subject to two rounds of heating to 95 °C for 5 min in TE followed by removal of the supernatant. Following this, the polymerase products bound to the beads were sequenced following the instructions from the kit using a primer labelled with C800. The conditions used for the reaction were as follows: anneal: 30 s at 52 °C, extension: 10 s at 70 °C and the reaction was carried out for 30 cycles. Amplified products were separated on 10% denaturing polyacrylamide gels and visualised using a Licor Odyssey scanner. Sequences were read manually.

## Conflict of interest

The authors declare no conflict of interest.

## Author contributions

SC and SK designed the experiments and wrote the paper. SC and GC carried out experiments.

### Peer review

The peer review history for this article is available at https://www.webofscience.com/api/gateway/wos/peer‐review/10.1111/febs.16936.

## Data Availability

The data that support the findings of this study are available in Figs 2, 3, 4, 5, 6, 7, 8, 9.

## References

[1] Bębenek A & Ziuzia‐Graczyk I (2018) Fidelity of DNA replication‐a matter of proofreading. Curr Genet 64, 985–996. 29500597 10.1007/s00294-018-0820-1 PMC6153641

[2] Burgers PMJ & Kunkel TA (2017) Eukaryotic DNA replication fork. Annu Rev Biochem 86, 417–438. 28301743 10.1146/annurev-biochem-061516-044709 PMC5597965

[3] Jain R , Rajashankar KR , Buku A , Johnson RE , Prakash L , Prakash S & Aggarwal AK (2014) Crystal structure of yeast DNA polymerase ε catalytic domain. PLoS One 9, e94835. 24733111 10.1371/journal.pone.0094835 PMC3986358

[4] Zheng F , Georgescu RE , Li H & O'Donnell ME (2020) Structure of eukaryotic DNA polymerase δ bound to the PCNA clamp while encircling DNA. Proc Natl Acad Sci USA 117, 30344–30353. 33203675 10.1073/pnas.2017637117 PMC7720213

[5] Yuan Z , Georgescu R , Schauer GD , O'Donnell ME & Li H (2020) Structure of the polymerase ε holoenzyme and atomic model of the leading strand replisome. Nat Commun 11, 3156. 32572031 10.1038/s41467-020-16910-5 PMC7308368

[6] Asturias FJ , Cheung IK , Sabouri N , Chilkova O , Wepplo D & Johansson E (2006) Structure of *Saccharomyces cerevisiae* DNA polymerase epsilon by cryo‐electron microscopy. Nat Struct Mol Biol 13, 35–43. 16369485 10.1038/nsmb1040

[7] Lancey C , Tehseen M , Raducanu VS , Rashid F , Merino N , Ragan TJ , Savva CG , Zaher MS , Shirbini A , Blanco FJ *et al*. (2020) Structure of the processive human pol δ holoenzyme. Nat Commun 11, 1109. 32111820 10.1038/s41467-020-14898-6 PMC7048817

[8] Zahurancik WJ , Klein SJ & Suo Z (2014) Significant contribution of the 3′→5′ exonuclease activity to the high fidelity of nucleotide incorporation catalyzed by human DNA polymerase ɛ. Nucleic Acids Res 42, 13853–13860. 25414327 10.1093/nar/gku1184 PMC4267634

[9] Albertson TM , Ogawa M , Bugni JM , Hays LE , Chen Y , Wang Y , Treuting PM , Heddle JA , Goldsby RE & Preston BD (2009) DNA polymerase epsilon and delta proofreading suppress discrete mutator and cancer phenotypes in mice. Proc Natl Acad Sci USA 106, 17101–17104. 19805137 10.1073/pnas.0907147106 PMC2761330

[10] Morrison A , Johnson AL , Johnston LH & Sugino A (1993) Pathway correcting DNA replication errors in *Saccharomyces cerevisiae* . EMBO J 12, 1467–1473. 8385605 10.1002/j.1460-2075.1993.tb05790.x PMC413358

[11] Rayner E , van Gool IC , Palles C , Kearsey SE , Bosse T , Tomlinson I & Church DN (2016) A panoply of errors: polymerase proofreading domain mutations in cancer. Nat Rev Cancer 16, 71–81. 26822575 10.1038/nrc.2015.12

[12] Barbari SR & Shcherbakova PV (2017) Replicative DNA polymerase defects in human cancers: consequences, mechanisms, and implications for therapy. DNA Repair (Amst) 56, 16–25. 28687338 10.1016/j.dnarep.2017.06.003 PMC5750057

[13] Pavlov YI , Zhuk AS & Stepchenkova EI (2020) DNA polymerases at the eukaryotic replication fork thirty years after: connection to cancer. Cancers (Basel) 12, 3489. 33255191 10.3390/cancers12123489 PMC7760166

[14] Xing X , Kane DP , Bulock CR , Moore EA , Sharma S , Chabes A & Shcherbakova PV (2019) A recurrent cancer‐associated substitution in DNA polymerase ε produces a hyperactive enzyme. Nat Commun 10, 374. 30670691 10.1038/s41467-018-08145-2 PMC6343027

[15] Shinbrot E , Henninger EE , Weinhold N , Covington KR , Göksenin AY , Schultz N , Chao H , Doddapaneni H , Muzny DM , Gibbs RA *et al*. (2014) Exonuclease mutations in DNA polymerase epsilon reveal replication strand specific mutation patterns and human origins of replication. Genome Res 24, 1740–1750. 25228659 10.1101/gr.174789.114 PMC4216916

[16] Barbari SR , Kane DP , Moore EA & Shcherbakova PV (2018) Functional analysis of cancer‐associated DNA polymerase ε variants in *Saccharomyces cerevisiae* . G3 (Bethesda) 8, 1019–1029. 29352080 10.1534/g3.118.200042 PMC5844290

[17] Ganai RA , Bylund GO & Johansson E (2015) Switching between polymerase and exonuclease sites in DNA polymerase ε. Nucleic Acids Res 43, 932–942. 25550436 10.1093/nar/gku1353 PMC4333401

[18] Pai CC & Kearsey SE (2017) A critical balance: dNTPs and the maintenance of genome stability. Genes (Basel) 8, E57. 10.3390/genes8020057 PMC5333046 28146119

[19] Traut TW (1994) Physiological concentrations of purines and pyrimidines. Mol Cell Biochem 140, 1–22. 7877593 10.1007/BF00928361

[20] Herzog M , Alonso‐Perez E , Salguero I , Warringer J , Adams DJ , Jackson SP & Puddu F (2021) Mutagenic mechanisms of cancer‐associated DNA polymerase ɛ alleles. Nucleic Acids Res 49, 3919–3931. 33764464 10.1093/nar/gkab160 PMC8053093

[21] Hodel KP , Sun MJS , Ungerleider N , Park VS , Williams LG , Bauer DL , Immethun VE , Wang J , Suo Z , Lu H *et al*. (2020) POLE mutation spectra are shaped by the mutant allele identity, its abundance, and mismatch repair status. Mol Cell 78, 1166–1177.e6. 32497495 10.1016/j.molcel.2020.05.012 PMC8177757

[22] Fang H , Barbour JA , Poulos RC , Katainen R , Aaltonen LA & Wong JWH (2020) Mutational processes of distinct POLE exonuclease domain mutants drive an enrichment of a specific TP53 mutation in colorectal cancer. PLoS Genet 16, e1008572. 32012149 10.1371/journal.pgen.1008572 PMC7018097

[23] Haradhvala NJ , Kim J , Maruvka YE , Polak P , Rosebrock D , Livitz D , Hess JM , Leshchiner I , Kamburov A , Mouw KW *et al*. (2018) Distinct mutational signatures characterize concurrent loss of polymerase proofreading and mismatch repair. Nat Commun 9, 1746. 29717118 10.1038/s41467-018-04002-4 PMC5931517

[24] Ganai RA & Johansson E (2016) DNA replication‐a matter of fidelity. Mol Cell 62, 745–755. 27259205 10.1016/j.molcel.2016.05.003

[25] Soriano I , Vazquez E , De Leon N , Bertrand S , Heitzer E , Toumazou S , Bo Z , Palles C , Pai CC , Humphrey TC *et al*. (2021) Expression of the cancer‐associated DNA polymerase ε P286R in fission yeast leads to translesion synthesis polymerase dependent hypermutation and defective DNA replication. PLoS Genet 17, e1009526. 34228709 10.1371/journal.pgen.1009526 PMC8284607

[26] Parkash V , Kulkarni Y , Ter Beek J , Shcherbakova PV , Kamerlin SCL & Johansson E (2019) Structural consequence of the most frequently recurring cancer‐associated substitution in DNA polymerase ε. Nat Commun 10, 373. 30670696 10.1038/s41467-018-08114-9 PMC6342957

[27] Bermudez VP , Farina A , Raghavan V , Tappin I & Hurwitz J (2011) Studies on human DNA polymerase epsilon and GINS complex and their role in DNA replication. J Biol Chem 286, 28963–28977. 21705323 10.1074/jbc.M111.256289 PMC3190704

